# Accuracy of zero-heat-flux thermometry and bladder temperature measurement in critically ill patients

**DOI:** 10.1038/s41598-020-78753-w

**Published:** 2020-12-10

**Authors:** Anselm Bräuer, Albulena Fazliu, Thorsten Perl, Daniel Heise, Konrad Meissner, Ivo Florian Brandes

**Affiliations:** 1 Department of Anesthesiology, University Medical Center Göttingen, Robert-Koch Strasse 40, 37099 Göttingen, Germany; 2 Department of General, Visceral and Pediatric Surgery, University Medical Center Göttingen, Göttingen, Germany

**Keywords:** Diagnosis, Physical examination

## Abstract

Core temperature (T_Core_) monitoring is essential in intensive care medicine. Bladder temperature is the standard of care in many institutions, but not possible in all patients. We therefore compared core temperature measured with a zero-heat flux thermometer (T_ZHF_) and with a bladder catheter (T_Bladder_) against blood temperature (T_Blood_) as a gold standard in 50 critically ill patients in a prospective, observational study. Every 30 min T_Blood_, T_Bladder_ and T_ZHF_ were documented simultaneously. Bland–Altman statistics were used for interpretation. 7018 pairs of measurements for the comparison of T_Blood_ with T_ZHF_ and 7265 pairs of measurements for the comparison of T_Blood_ with T_Bladder_ could be used. T_Bladder_ represented T_Blood_ more accurate than T_ZHF_. In the Bland Altman analyses the bias was smaller (0.05 °C vs. − 0.12 °C) and limits of agreement were narrower (0.64 °C to − 0.54 °C vs. 0.51 °C to – 0.76 °C), but not in clinically meaningful amounts. In conclusion the results for zero-heat-flux and bladder temperatures were virtually identical within about a tenth of a degree, although T_ZHF_ tended to underestimate T_Blood_. Therefore, either is suitable for clinical use.

German Clinical Trials Register, DRKS00015482, Registered on 20th September 2018, http://apps.who.int/trialsearch/Trial2.aspx?TrialID=DRKS00015482.

## Introduction

Core temperature monitoring is one of the essential monitoring modalities in intensive care medicine and temperatures below or above the normal core temperature can be observed frequently. Hypothermia at admission of surgical patients is associated with many adverse outcomes like coagulopathy^1^, increased bleeding^2^, and higher transfusion rates^3^ as well as increased surgical site infections^4,5^ and in some studies even with mortality^3,6–9^. However, fever is observed much more frequently in critically ill patients and warrants a diagnostic workup to determine the presence of potential infections^10^. The ideal temperature measurement method should provide reliable, reproducible values safely and conveniently^10^. Additionally, the device should be small, easy to use, comfortable, fast, continuous, noninvasive, low energy consuming and affordable^11^. In many institutions bladder temperature (T_Bladder_) is the standard of care because it is accurate, provides continuous readings, stable measurements regardless of urine flow rate and stays securely in place even during positioning of the patient^10^. Normally T_Bladder_ monitoring adds no additional invasiveness to the standard monitoring, because bladder catheters are nearly always used in critically ill patients.

However, not every patient meets the criteria for a bladder catheter. Patients with acute or chronic renal failure with anuria, after cystectomy or awake patients do not need a bladder catheter. With no indication for using a bladder catheter the use is associated with an unnecessary risk of nosocomial infection. Further on, patients that need irrigation of the bladder because of bleeding will not be appropriate for measurement of T_Bladder_. In these situation an alternative approach is needed. Especially in alert patients a non-invasive monitor is helpful. Unfortunately, many of these methods are not very reliable^12^. A better alternative might be a zero-heat flux (ZHF) thermometer that has been proven to be reliable in surgical patients^13–16^. In general, zero-heat-flux thermometers consist of a thermal insulator adjacent to the skin that is covered by a servo-controlled electric heater. The heater is used to eliminate the flow of heat through the insulator, so that the temperature of the heater and skin temperatures are equal^13^. A validation study in critically ill patients is important because in patients undergoing surgery the most common thermal problem is perioperative hypothermia whereas in critically ill patients it is fever. However, to date no large comparison in critically ill patients has been performed with an accepted gold standard like blood temperature. There is only one study available that has included a small number of patients with blood temperature as a reference method^26^.

The aim of this study was to compare T_Core_ measured with a ZHF-thermometer (T_ZHF_) and with T_Bladder_ against a gold standard T_Blood_ measured in the iliac artery or pulmonary artery to determine if the new ZHF-thermometer is more accurate than T_Bladder_.

## Methods

The current prospective clinical study was conducted in accordance with the declaration of Helsinki at the University Hospital of Göttingen, Germany, after obtaining local ethics committee approval (Ethics committee of the University Medicine Göttingen, Application number: 13/05/18) for the experimental protocol and registration on German Clinical Trials Register (DRKS00015482). According to the approval of the local ethical committee we used deferred (proxy) consent in emergency critical care research^17^ as the study was totally non-invasive and observative. If patients were able to give informed written consent this consent was used. If informed proxy consent was necessary, it was given in written form of the proxy. We did not exclude patients who did not recover and died during their hospital stay. The local ethics committee had approved this procedure. The article adheres to the STROBE guidelines^18^.

Critically ill adult patients already having a bladder catheter with a temperature probe and an arterial catheter with a temperature probe placed in the iliac artery (Pulsiocath Arterial Thermodilution Catheter 5F; Pulsion Medical Systems AG, Munich, Germany) or a pulmonary artery catheter (Arrow Hands-Off Thermodilution catheter 7F; Arrow International, Athlone, Irland) in place were included in this this study. The only exclusion criteria were pregnancy and refusal to take part in the study.

In all patients, core temperature was measured additionally with a single use, continuous, non-invasive ZHF-thermometer (3 M SpotOn Temperature Monitoring System, 3 M, St. Paul, MN, USA) attached to the lateral forehead of the patients.

Then every 30 min T_Core_ measured by T_Blood_, T_Bladder_ and the ZHF-thermometer were documented at the same time points until the patient lost the T_Blood_ sensor or T_Bladder_ sensor, left the ICU or at least after 5 days. If data of a temperature source were missing the couple of data was not used for comparison. In addition to the temperature data age, weight, height, sex and medical diagnosis at admission to the Intensive Care Unit (ICU) were documented.

As a primary statistical method Bland–Altman statistics were used for interpretation of accuracy (bias = mean difference between methods) and precision (limits of agreement = 1.96 standard deviation) using the Bland and Altman random effects method for repeated measures data adjusted for unequal numbers of measurements per patient^19^. Additionally, we calculated the proportion of all differences that were within ± 0.5 °C or ± 1 °C of T_Blood_.

For each of the two measurement modalities sensitivity, specificity, positive and negative predictive values for the detection of hypothermia and fever were calculated. Hypothermia was defined as a T_Blood_ < 36 °C and fever was defined as T_Blood_ > 38.3 °C^10^.

Additionally, we performed an error grid analysis^20^ to determine if some measurement differences would lead to wrong clinical decisions. The Zones were defined as follows:

Zone A begins with an area of a ± 0.5 °C error on either side of a perfectly accurate measurement between T_Blood_ and the temperature measured by T_ZHF_ or T_Bladder_. Measurement errors smaller than ± 0.5 °C are considered by most authors as clinically not relevant. In addition, if both measurements indicate hypothermia < 36 °C or fever > 38.3 °C the absolute error is considered to be clinically irrelevant because the same treatment or diagnostic workup will be initiated.

Zone B describes the zone where measurement errors are > 0.5 °C but this will not result in a clinical wrong decision. E.g. if T_Blood_ is 36.5 °C and T_ZHF_ shows a temperature of 37.4 °C both temperatures will not lead to active warming therapy or a diagnostic workup for infection.

In contrast Zone C indicates errors larger than 0.5 °C that will lead to wrong clinical decisions and may do harm to the patient. e.g. if T_Blood_ is 34 °C and T_ZHF_ shows 37 °C the patient will not receive active warming although this would be indicated.

## Results

55 potentially eligible patients were screened. Three patients could not be enrolled because we could not obtain proxy consent and two patients were not included due to technical problems. The remaining 50 patients were enrolled. 36 patients (72%) were male, 14 (28%) were female. Mean age was 61.9 (± 16.8) years, mean height was 1.75 (± 0.07) m, mean weight was 86.4 (± 36.3) kg resulting in a mean body mass index of 28.2 (± 11.3) kg/m^2^. Of these patients 16 were suffering from sepsis, 18 patients had neurologic injury (subarachnoid hemorrhage, intracerebral hemorrhage), 6 patients had trauma, 4 patients had respiratory failure, 2 patients had accidental hypothermia, 3 patients had cardiac surgery, and 1 patient had visceral surgery. Of all 50 patients 49 had an arterial catheter with a temperature probe placed in the iliac artery and one patient had a pulmonary artery catheter with temperature probe. No patient was excluded from the study after enrolment.

Globally 3970.5 h were recorded. 7665 T_Blood_ values, 7086 values of T_ZHF_ and 7358 T_bladder_ values were documented. 276 T_Blood_ values, 855 values of the ZHF-thermometer and 583 T_Bladder_ values were missing. The major reason for missing values was a disconnection of the temperature probes for transportation of the patient to the CT, OR, neuroradiology suite, or to the cardiac catheter lab. After these procedures the devices were often not reconnected immediately. Only 17 temperature values of T_Bladder_ and 16 values of T_ZHF_ were missing due to technical problems. 12 values below 30 °C could not be recorded by the ZHF-thermometer because the device did not give a reading at these low temperatures.

This resulted in 7018 pairs of measurements for the comparison of T_Blood_ with T_ZHF_ and 7265 pairs of measurements for the comparison of T_Blood_ with T_Bladder_.

In 530 measurements T_Blood_ was < 36 °C, in 6665 measurements T_Blood_ was 36–38.3 °C and in 470 measurements T_Blood_ was > 38.3 °C.

### Bland Altman analysis

**Figure 1:**
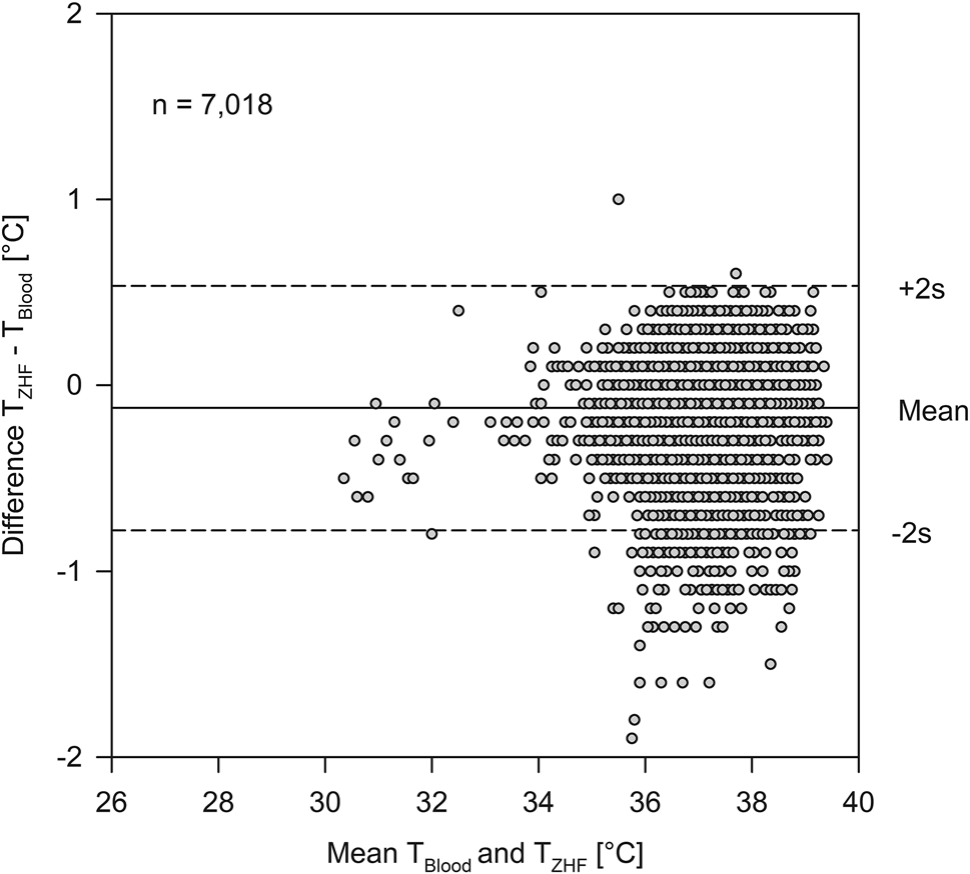
Bland–Altman analysis for the zero-heat flux thermometer (T_ZHF_) versus blood temperature (T_Blood_).

**Figure 2:**
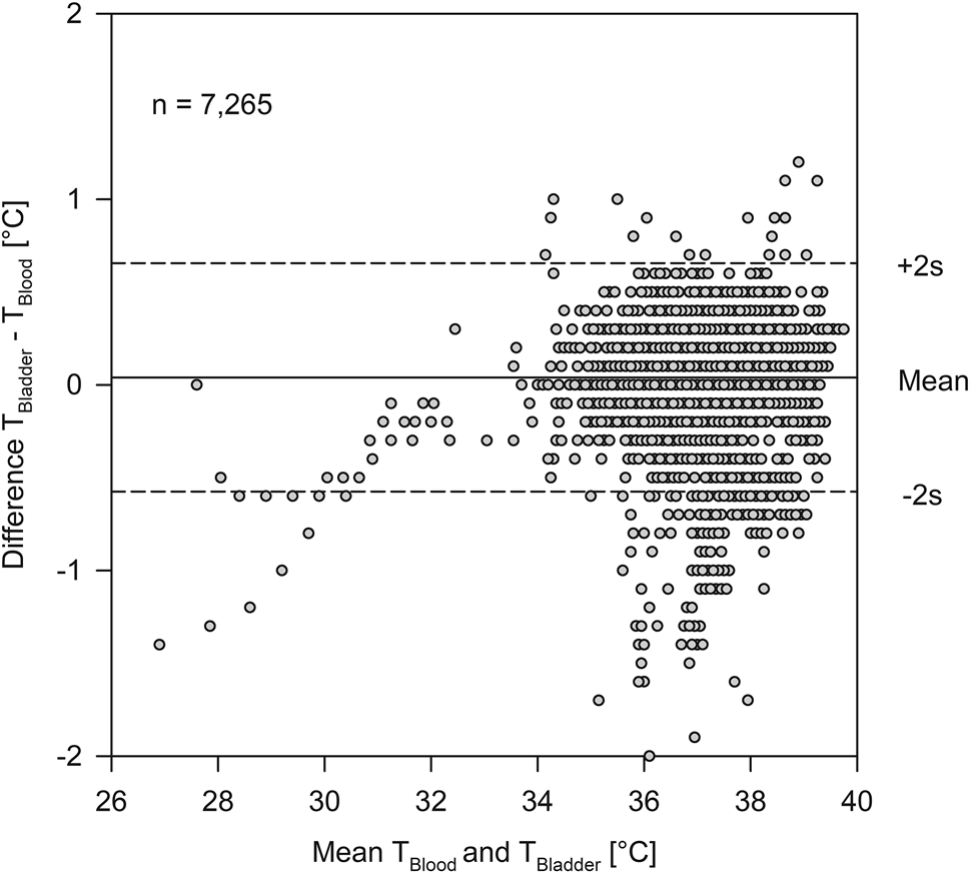
Bland–Altman analysis for bladder temperature (T_Bladder_) versus blood temperature (T_Blood_).

Bias between T_ZHF_ and T_Blood_ was − 0.12 °C with an upper limit of agreement of 0.51 °C and a lower limit of agreement of − 0.76 °C (Fig. 1). Bias between T_Bladder_ and T_Blood_ was 0.05 °C with an upper limit of agreement of 0.64 °C and a lower limit of agreement of − 0.54 °C (Fig. 2).

### Proportion of differences within ± 0.5 °C and ± 1 °C

The proportion of differences within ± 0.5 °C of T_Blood_ was 90.98% for T_ZHF_ and 95.99% for T_Bladder_ and the proportion of differences within ± 1.0 °C of T_Blood_ was 98.99% for T_ZHF_ and 99.01% for T_Bladder_.

### Sensitivity, specificity, positive and negative predictive values

The calculated sensitivity, specificity, positive and negative predictive values for the detection of hypothermia and fever are shown in Table 1.

**Table 1:** Sensitivity, specificity, positive and negative predictive values for the detection of hypothermia and fever of both methods.

	Sensitivity [%]	Specificity [%]	PPV [%]	NPV [%]
**Detection of hypothermia**				
T_ZHF_	0.89	0.96	0.62	0.99
T_Bladder_	0.81	0.99	0.84	0.96
**Detection of fever**				
T_ZHF_	0.65	0.98	0.74	0.97
T_Bladder_	0.83	0.97	0.67	0.98

*T*_*ZHF*_ temperature measured with a zero-heat flux thermometer, *T*_*Bladder*_ bladder temperature, *PPV* positive predictive value, *NPV* negative predictive value.

### Error grid analysis

**Figure 3:**
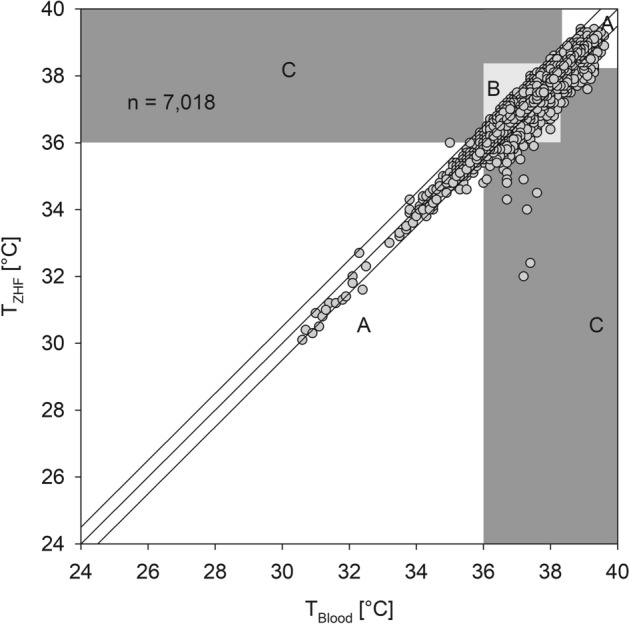
Error grid analysis of the zero-heat flux thermometer (ZHF) against blood temperature (T_Blood_). Zone A is drawn in white, Zone B in grey and Zone C in dark grey.

**Figure 4:**
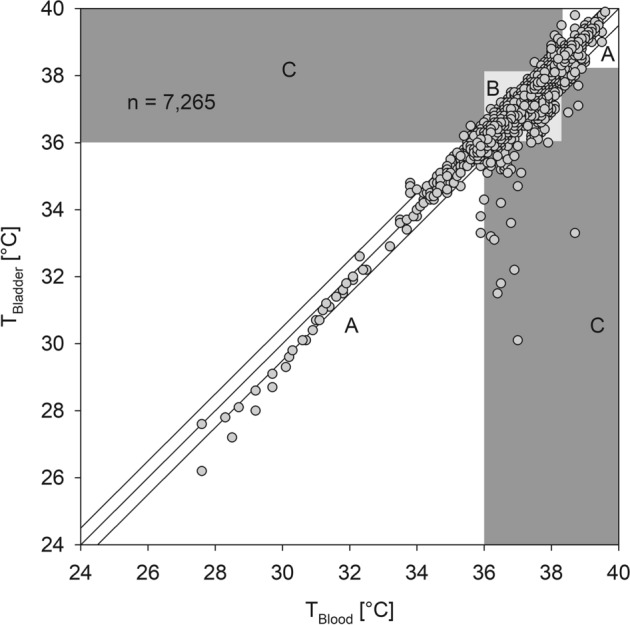
Error grid analysis of bladder temperature (T_Bladder_) against blood temperature (T_Blood_). Zone A is drawn in white, Zone B in grey and Zone C in dark grey.

Error grid analysis showed that 91.6% of all T_ZHF_ measurements were clinically not different from T_Blood_, or would still lead to the same treatment or diagnostic workup. In 6.2% measurement errors were > 0.5 °C, but the result would not lead to a clinical wrong decision. Only 2.2% of the measurements would lead to wrong clinical decisions (Fig. 3). Error grid analysis of T_Bladder_ showed that 96.3% of all measurements were clinically not different from T_Blood_ or would still lead the same treatment or diagnostic workup. In 2.4% measurement errors were > 0.5 °C but this would not result in a clinical wrong decision. Only 1.3% of the measurements would lead to wrong clinical decisions (Fig. 4).

### Adverse events

The ZHF-thermometer sensors were well tolerated in all patients and no burn or skin reaction was observed during the study period.

## Discussion

In this study with critically ill patients, T_Bladder_ represented T_Blood_ more accurate than T_ZHF_. In the Bland Altman analyses the bias was smaller and limits of agreement were narrower. The proportion of differences within ± 0.5 °C of T_Blood_ were higher, and there were less values in Zone B and C of the error grid analysis. In addition, the ZHF thermometer failed to record core temperatures below 30 °C. However, compared to the published results for other non-invasive thermometers like infrared tympanic membrane thermometers, temporal artery thermometers, or axillary thermometers^12^ the ZHF-thermometer is more accurate.

### Interpretation of our results

The results of the Bland Altman analysis of T_Bladder_ were comparable to the results that were obtained by Nierman^21^ and slightly different from the results of Lefrant et al.^22^ who observed a bias of − 0.21 °C and more narrow limits of agreement of ± 0.20 °C. In general, the high level of accuracy of T_Bladder_ is remarkable because oliguria, that is very frequent in ICU patients, reduces the accuracy of T_Bladder_ measurements in operative patients^23,24^. On the other hand, in critically ill patients, oliguria does not seem to influence the accuracy of bladder temperature very much^10^.

The results of the Bland Altman analysis of the ZHF-thermometer were a slightly better than the results that were obtained by Eshraghi et al.^13^ before and after cardiopulmonary bypass, and in the same range as found by Mäkinen et al.^15^ during cardiac surgery when the patients were off cardiopulmonary bypass. During surgery with slow temperature changes Boisson et al.^14^ could obtain better results with a bias to T_Blood_ of − 0.1 °C with limits of agreement of ± 0.4 °C.

The proportion of differences within ± 0.5 °C of T_Blood_ was 84% in the study of Eshraghi^13^ and 94% in the study of Boisson^14^. Our results of 91% are also in that range. Other studies that have evaluated the ZHF-thermometer in critically ill patients did not compare it to a gold standard and are therefore of limited value for the comparison with our results^25,26^.

The question is, if the accuracy of the ZHF-thermometer is still good enough to be used in ICU. Many studies that compare new temperature monitoring devices with a gold standard use a definition that the combined inaccuracy (bias and limits of agreement) should be smaller than 0.5 °C^27^ to be accurate enough. In our opinion this objective is very high and most of the studies that have investigated new non-invasive thermometers^13,28,29^ did not find an accuracy that met this criterion. Still they came to the conclusion that the new devices agree sufficiently enough for clinical practice^13,28,29^.

Another possibility is to look at the proportion of differences within the range of ± 0.5 °C of the T_Blood_. In our study 91% of all measurement values of the ZHF-thermometer were within the range of ± 0.5 °C of T_Blood_ and 99% were within the range of ± 1 °C. That seems to be acceptable.

Another interesting way of interpreting the results is the error grid analysis^20^. In this analysis 91.6% of the values of the ZHF-thermometer lead to the right clinical decision and only 2.2% of the measured values would lead to wrong clinical decisions. This seems to be sufficient, especially because T_Core_ changes do not require an immediate change in therapy in the next minutes. However, it has been argued, that no single measurement value should be in Zone C as this will lead to wrong clinical decisions^20^. This seems to be very demanding. If we would accept this, methods like non-invasive blood pressure measurement or pulse oximetry would have to be abandoned immediately.

## Limitations of the study

In most studies comparing temperature measurement devices there are many data pairs per subject and the number of data pairs per patient are not equal. This can induce random effects because there are independent influences of the different patients and there are influences of time in each individual patient. This influence is not totally independent. To account for this effect, we have used the Bland and Altman random effects method for repeated measures data adjusted for unequal numbers of measurements per patient^19^.

A potential limitation of the methods used is the use of error grid analysis. This method has not been used for the comparison of temperature measurement devices before. Error grid analysis is highly dependent on the zones, which can are by definition arbitrarily defined. In this study the zones were defined by the authors a priori using published and well accepted definitions. Zone A was defined as an area of a ± 0.5 °C error on either side of a perfectly accurate measurement between T_Blood_ and the T_ZHF_ or T_Bladder_ because measurement errors smaller than ± 0.5 °C are considered by most authors as clinically not relevant. In addition, if both measurements indicate hypothermia < 36 °C or fever > 38.3 °C the absolute error is considered to be clinically irrelevant because the same treatment or diagnostic workup will be initiated. It can be argued that there is a clinically relevant difference between 35.0 °C and 26 °C. This would still lead to a data point that is in the Zone A. However, it is extremely difficult to define thresholds for this situation. In addition, we did not observe this.

Other potential limitations of our study are that we have studied a relatively small population with only 50 patients. However, in average every patient was monitored more than 3.3 days, resulting in an average of about 140 measurement points per patient.

Another potential limitation is that we have studied a mixed ICU patient collective. This may also be seen as an advantage because we have measured different patients in different critically ill states and with different influences like renal replacement therapy (RRT) or Extracorporeal Membrane Oxygenation (ECMO). Patients undergoing targeted temperature management after cardiopulmonary resuscitation which might be an interesting and challenging patient cohort in which T_Core_ measurement is of utmost importance are missing in our collective. This may be a limitation to the generalizability of the study results.

In some of our patients the gold standard blood temperature may be distorted by the rapid infusion of unwarmed fluids or extracorporeal devices like RRT or ECMO. It is well known that a rapid infusion of unwarmed or cold fluids can lower blood temperature temporarily. This effect is typically used for the measurement cardiac output with a pulmonary artery catheter. This effect varies depending on the temperature, amount, and rate of the fluid given. Initiation of RRT also temporarily changes blood temperature to a small amount but a stable running RRT does not lead to changes in blood temperature. The same is probably true for ECMO. Infusion of intravenous fluids or RRT are typical measures in ICU and it is not possible to exclude patients that need intravenous fluids. In our patient group 17 patients had RRT and 2 patients had ECMO. This may have contributed to the observed inaccuracy of the ZHF-thermometer and T_Bladder_. Another potential problem may be that the analogue data transfer from the ZHF-thermometer to the general ICU monitoring may have introduced an additional error.

We did also not observe many measurements for temperature above 39 °C. Therefore, it is not possible to make any conclusions about the accuracy the devices in these extremely high temperature range.

The use of vasopressor therapy and especially the use of high dose vasopressor therapy may also influence the accuracy of the ZHF-thermometer. Unfortunately, we did not look at this potential source of inaccuracy. This might be investigated in another study.

Also we did not measure the urine output of our patients, therefore a correlation to accuracy of T_Bladder_ is impossible.

Some studies have used more complex statistical methods^29^ like population analysis^30^. However, very often these complex analyses do not add very much new information about the accuracy of the studied devices. We included sensitivity, specificity, positive and negative predictive values for the detection of hypothermia and fever for both methods because this has not been done yet. We also included an error grid analysis because this kind of analysis may be clinically useful although the definition of the three zones in that error grid analysis can be discussed.

## Conclusion

In conclusion the results for zero-heat-flux and bladder temperatures were virtually identical within about a tenth of a degree, although T_ZHF_ tended to underestimate T_Blood_. Therefore, either is suitable for clinical use and can be used if bladder temperature is not available.

## Data Availability

The datasets used for the analysis in the current study are available from the corresponding author on reasonable request.
